# Congenital syphilis in Portugal, 2015–2024: Temporal-spatial characterization and gaps in prevention of vertical transmission

**DOI:** 10.1017/S095026882610140X

**Published:** 2026-04-17

**Authors:** Filipa Canha, Sebastian von Schreeb, Pedro Parreira da Silva, Inês Martins, Ana Mendes, Dina Oliveira, Pedro Pinto Leite, Vítor Cabral Veríssimo

**Affiliations:** 1 Public Health Service, Almada-Seixal Local Health Unit, Portugal; 2 Center for Public Health Emergencies, Directorate-General of Health, Portugal; 3 Directorate of Information and Analysis, Directorate-General of Health, Portugal; 4 ECDC Fellowship Programme, Field Epidemiology path (EPIET), European Centre for Disease Prevention and Control, Sweden; 5 Unidade de Saúde Familiar do Parque, Santa Maria Local Health Unit, Portugal; 6 Directorate of Disease Prevention and Health Promotion, Directorate-General of Health, Portugal; 7 Department of Obstetrics and Gynecology, Santa Maria University Hospital, Local Health Unit of Santa Maria, Lisbon, Portugal; 8 Clínica Universitária de Obstetrícia e Ginecologia, Universidade de Lisboa Faculdade de Medicina, Portugal; 9 Instituto de Medicina Preventiva e Saúde Pública (IMPSP), Universidade de Lisboa Faculdade de Medicina, Portugal; 10 Public Health Unit, Western Lisbon Local Health Unit, Portugal

**Keywords:** antenatal care, congenital syphilis, surveillance, vertical transmission, elimination, prevention

## Abstract

Aligned with the increasing trend observed across the EU/EEA, congenital syphilis (CS) cases have risen in Portugal, which has the third highest rate per 100000 live births in the EU/EEA. This study aimed to analyse CS cases reported in Portugal, focusing on pregnancy monitoring and antenatal screening to identify gaps in preventing vertical transmission of syphilis. We conducted a descriptive study, including confirmed CS cases reported in Portugal from 2015 to 2024. We calculated annual incidence per 100000 live births and the proportion of pregnancies monitored and antenatal screenings performed. During 2015–2024, 99 confirmed CS cases were reported, 64.6% in infants under 1 month of age. The incidence of CS increased eightfold from 2016 to 2024. Among mothers of CS cases, 67.7% had pregnancies classified as monitored; of these, 77.6% had a record of antenatal screening, and 88.5% of those screened tested positive. These findings highlight potential fragilities in antenatal care, diagnosis and treatment, contributing to the resurgence of CS in Portugal. Addressing missed opportunities for prevention requires improving maternal healthcare, strengthening surveillance systems, and ensuring the timely treatment of pregnant people and their partners, in order to reverse this trend and move towards the elimination of vertical transmission of syphilis.

## Introduction

Syphilis is a sexually transmitted infection (STI) caused by the bacterium *Treponema pallidum* (TP). Clinical manifestations emerge after an incubation period of 10 to 90 days. Syphilis progresses through three stages (primary, secondary, and tertiary) and may include an asymptomatic period during the infection (latent syphilis) [1].

Syphilis is primarily transmitted sexually via direct contact with infected lesions or bodily fluids, including blood, or blood-derived products, but can also be transmitted vertically [1]. Although vertical transmission of syphilis can occur at any stage of pregnancy, the risk increases with gestational age [2–4]. Pregnant people diagnosed with primary and secondary syphilis face vertical transmission rate of 60 to 100%, whereas in the late latent stage, the risk is below 8% [1].

Vertical transmission of syphilis can lead to foetal or perinatal death, prematurity, foetal grow restriction, low birth weight, and congenital syphilis (CS) if not properly identified and treated [4–6]. CS may be asymptomatic or present with hepatosplenomegaly, jaundice, maculopapular rash, syphilitic rhinitis, bone abnormalities, central nervous system involvement, nephrotic syndrome, or haematological changes. If left untreated, it can lead to long-term complications affecting bones, teeth, eyes, and skin [1, 3, 4].

Antenatal screening and penicillin treatment for pregnant people diagnosed with syphilis are among the most cost-effective public health interventions available, significantly reducing the burden of CS [7, 8]. In Portugal, universal syphilis screening is recommended in both first and third trimesters of pregnancy using the Venereal Disease Research Laboratory (VDRL) test, although different screening approaches are adopted in other countries [9–11]. A reactive VDRL test should be confirmed by a treponemal test, such as *T. pallidum* Hemagglutination Assay (TPHA) or Fluorescent Treponemal Antibody Absorption test (FTA-Abs) [9, 10]. Treatment is indicated for all pregnant people diagnosed, irrespective of the stage of infection [12]. When administered at least 30 days before delivery-typically with benzathine penicillin-effectively cures the maternal infection and prevents congenital transmission to the foetus [12]. Treatment is also indicated for newborns diagnosed with CS, and for newborns whose mothers were untreated or received treatment within 30 days prior to delivery [12]. Proper treatment during pregnancy can reduce stillbirth by up to 82%, incidence of prematurity by 64%, and neonatal death by more than 90% [13].

Aligned with the EU, syphilis and CS are mandatory notifiable diseases in Portugal [14]. The incidence of reported CS in the European Union/European Economic Area (EU/EEA) has fluctuated over the past decade but has shown an increasing trend in recent years [15]. In 2023, Portugal reported the highest absolute number of CS cases among EU/EEA (*n* = 14) countries and ranked third as the country with the highest reporting rate, with 16.3 cases per 100000 live births [15]. This highlights the need to identify gaps in the prevention of vertical transmission of syphilis to develop effective strategies for CS elimination.

The World Health Organization (WHO) has established criteria to validate the elimination of CS, which require a CS case rate of fewer than 50 cases per 100000 live births and at least 95% coverage of antenatal care, screening, and treatment of syphilis during pregnancy [16]. The WHO Regional Office for Europe has also set elimination targets, aiming to reduce CS incidence to 10 cases per 100000 live births or fewer by 2025, and 1 case per 100000 live births or fewer by 2030 [17].

The study aims to characterize confirmed CS cases reported in Portugal between 2015 and 2024 in terms of temporal and spatial distribution, clinical presentation, maternal demographic profile, and antenatal care history. The frequency of pregnancy monitoring and antenatal screening in these cases will also be assessed to identify gaps and improve prevention strategies.

## Methods

A retrospective, descriptive study was conducted, including all confirmed cases of CS reported through the support platform of the National Epidemiological Surveillance System (SINAVE) between 2015 and 2024. All cases were classified according to the EU case definition [18].

A confirmed case of CS was defined as any infant who meets the laboratory criteria for a confirmed case, without the need to meet the clinical criteria. The laboratory criteria for a confirmed case requires at least one of the following: (i) demonstration of TP by dark field microscopy in the umbilical cord, the placenta, a nasal discharge, or skin lesion material, (ii) demonstration of TP by direct fluorescent-antibody testing for TP (DFA-TP) in the umbilical cord, the placenta, a nasal discharge, or skin lesion material, (iii) and detection of TP-specific IgM (FTA-Abs, enzyme immunoassay [EIA]) and a reactive non-treponemal test (VDRL, Rapid Plasma Reagin [RPR]) in the child’s serum [14, 18].

Data on annual live births was obtained from the Statistics Portugal database [19], disaggregated by Nomenclature of Territorial Units for Statistics (NUTS) level I region.

The analysis included demographic and clinical variables related to the newborn, demographic and risk-factor variables related to the mother, and variables related to antenatal care. Injecting drug use (IDU) was included because it is a recognized risk factor for STI acquisition, including syphilis, and may reflect social vulnerability associated with delayed antenatal care and missed screening opportunities.

We calculated relative frequencies for categorical variables. The annual incidence of CS per 100000 live births was calculated for Portugal as a whole and for each NUTS I region. These incidence rates were compared with the WHO/Europe targets for the elimination of vertical transmission of syphilis for 2025 and 2030 [17].

We calculated the proportion of confirmed CS cases in which the pregnancy was monitored. A pregnancy was considered monitored if, pursuant to the provisions of the National Program for Monitoring Low-Risk Pregnancy [10] and Standard No. 001/2023 (27 January) [20], issued by the Directorate-General of Health, the attending physician who reported the case formally reported it as such. This approach reflects the clinical judgement documented in the official notification form rather than an independent assessment conducted within this study. Among the monitored pregnancies, the proportion in which antenatal screening was performed in the first and third trimesters was calculated, along with the proportion of reactive results in each screening period.

Ethical approval was not required because the study used anonymized national surveillance data accessed under the legal mandate of the Portuguese National Health Authority for public-health purposes.

## Results

Between 2015 and 2024, 99 confirmed CS cases were reported in Portugal, corresponding to a mean annual incidence of 11.7 CS cases per 100000 live births. The characteristics of the confirmed cases are summarized in Table 1. Of these, 64.6% were infants under one month of age and 34.3% were between 1 and 12 months old at time of reporting; 52.5% occurred in males. Symptoms were recorded in 51.5% of cases. Among symptomatic infants, the most frequently reported manifestations were jaundice (29%), hepatosplenomegaly (24%) and anaemia (23%). No deaths were reported among the confirmed cases. In terms of geographic distribution, based on the infant’s region of residence, most cases occurred in mainland Portugal (90.3%) (Table 1).

**Table 1.** Demographic profile of confirmed congenital syphilis cases, Portugal, 2015–2024 Table 1. long description.

Variables	Number of confirmed CS cases (%)(*N* = 99)^a^
**Sex**	
Male	52 (52.5%)
Female	47 (47.5%)
**Age group**	
<1 month	64 (64.6%)
1–12 months	34 (34.3%)
>12 months	1 (1.0%)
**NUTS I region**	
Mainland	84 (90.3%)
Azores AR	9 (9.7%)
Madeira AR	0 (0.0%)
Symptoms* *Total*	
Yes	51 (51.5%)
No	45 (45.5%)
Unknown	3 (3.0%)
*<1 month of age*	
Yes	30 (46.9%)
No	31 (48.4%)
Unknown	3 (4.69%)
*1–12 months of age*	
Yes	20 (58.8%)
No	14 (41.2%)
Unknown	0 (0%)
*>12 months of age*	
Yes	1 (100%)
No	0 (0%)
Unknown	0 (0%)
*Jaundice*	
Yes	29 (29.3%)
No	67 (67.7%)
Unknown	3 (3.0%)
*Hepatosplenomegaly*	
Yes	24 (24.2%)
No	70 (70.7%)
Unknown	4 (4.0%)
*Anaemia*	
Yes	23 (23.2%)
No	70 (70.7%)
Unknown	6 (6.1%)
*Mucocutaneous lesions*	
Yes	18 (18.2%)
No	75 (75.8%)
Unknown	6 (6.1%)
*Central nervous involvement*	
Yes	15 (15.2%)
No	72 (72.7%)
Unknown	12 (12.1%)
*Pseudoparalysis (due to periostitis and osteochondritis)*	
Yes	4 (4.0%)
No	89 (89.9%)
Unknown	6 (6.1%)
*Persistent rhinitis*	
Yes	3 (3.0%)
No	90 (90.9%)
Unknown	6 (6.1%)
*Malnutrition*	
Yes	3 (3.0%)
No	90 (90.9%)
Unknown	6 (6.1%)
*Condyloma lata*	
Yes	1 (1.0%)
No	91 (91.9%)
Unknown	7 (7.1%)
*Nephrotic syndrome*	
Yes	0
No	93 (93.9%)
Unknown	6 (6.1%)
**Demonstration of *Treponema pallidum* by dark field microscopy in the umbilical cord, the placenta, a nasal discharge, or skin lesion material**	
Yes	3 (3.0%)
No	85 (85.9%)
Unknown	11 (11.1%)
**Demonstration of *T. pallidum* by DFA-TP in the umbilical cord, the placenta, a nasal discharge, or skin lesion material**	
Yes	1 (1.0%)
No	85 (85.9%)
Unknown	13 (13.1%)
**Detection of *T. pallidum*-specific IgM and a reactive non-treponemal test in the child’s serum**	
Yes	97 (98.0%)
No	0 (0.0%)
Unknown	2 (2.0%)

*Note*: AR, Autonomous Regions; CS, Congenital Syphilis; DFA-TP, Direct Fluorescent Antibody *T. pallidum*; IgM, Immunoglobulin M; NUTS, Nomenclature of Territorial Units for Statistics *At least one of the symptoms to fulfil clinical criteria according to the 2018 EU case definition: hepatosplenomegaly, mucocutaneous lesions, condyloma lata, persistent rhinitis, jaundice, pseudoparalysis (due to periostitis and osteochondritis), central nervous involvement, anaemia, nephrotic syndrome, or malnutrition [18].

a *n* (%).

Regarding maternal age at the time of delivery, most mothers were aged 20 to 29 years (40.4%) or 30 to 39 years (31.3%). In 13.1% of cases, the maternal age was unknown or not reported by the notifying physician (Table 2).

**Table 2.** Profile of the mothers of confirmed congenital syphilis cases, Portugal, 2015–2024 Table 2. long description.

Variable	Number of confirmed CS cases (%)(*N* = 99)^a^
**Mothers’ age group**	
<20 years	11 (11.1%)
20–29 years	40 (40.4%)
30–39 years	31 (31.3%)
>40 years	4 (4.0%)
Unknown	13 (13.1%)
**Mothers’ nationality**	
Portugal	48 (48.5%)
Foreign	21 (21.2%)
Unknown	30 (30.3%)
**Syphilis stage**	
Primary	13 (13.1%)
Secondary	3 (3.0%)
Early latent	16 (16.2%)
Late latent	12 (12.1%)
Unknown	55 (55.6%)
**Previously given birth to a child diagnosed with CS**	
Yes	2 (2.1%)
No	70 (72.2%)
Unknown	25 (25.8%)
**Ongoing IDU**	
Yes	1 (1.0%)
No	63 (65.6%)
Unknown	32 (33.3%)
**Previous history of IDU**	
Yes	3 (3.1%)
No	59 (61.5%)
Unknown	34 (35.4%)

*Note*: CS, congenital syphilis, IDU, injecting drug use.

a *n* (%).

Over the 10-year period, maternal nationality was reported as Portuguese in 48.5% of confirmed CS cases and foreign in 21.2%, while in 30.3% of cases this information was not reported. Between 2015 and 2019, maternal nationality was not reported in 85.2% of confirmed cases, and from 2020 to 2024, the mother’s nationality was Portuguese in 62.5% of confirmed cases and foreign in 29.2%.

A previous history of maternal IDU was recorded in 3.0% of confirmed cases. In addition, one case involved ongoing IDU by the mother during pregnancy. Latent syphilis was the most frequently reported stage of maternal infection at the time of diagnosis (28.3%). However, in the majority of CS cases (55.6%), the maternal stage of infection remained unknown. Of note, in two cases, the mothers had previously given birth to a child diagnosed with CS.

**Figure 1.**
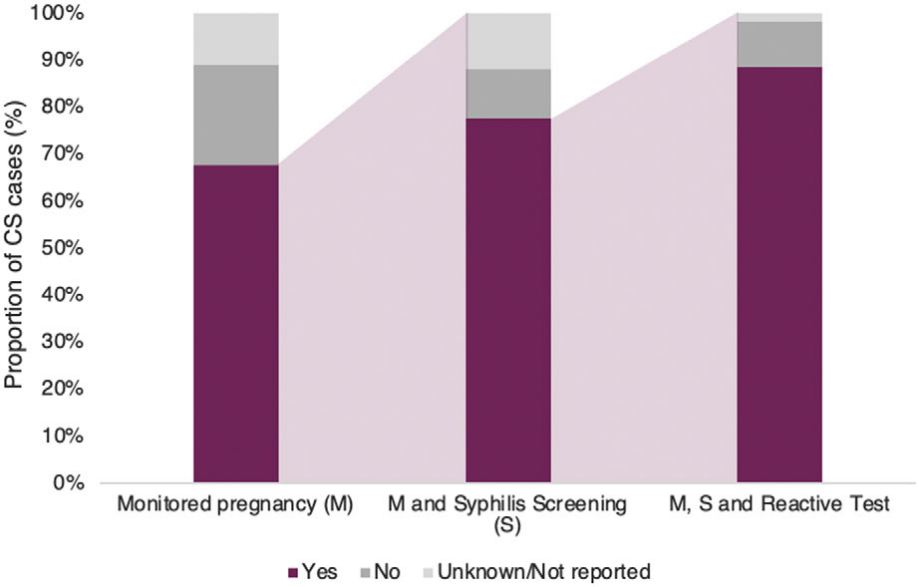
Proportion of confirmed congenital syphilis cases with monitored pregnancies (M); the subset of these cases that underwent antenatal syphilis screening in the 1st and 3rd trimesters (S), and the proportion of these with a reactive test result, from 2015 to 2024. Figure 1. long description.

**Figure 2.**
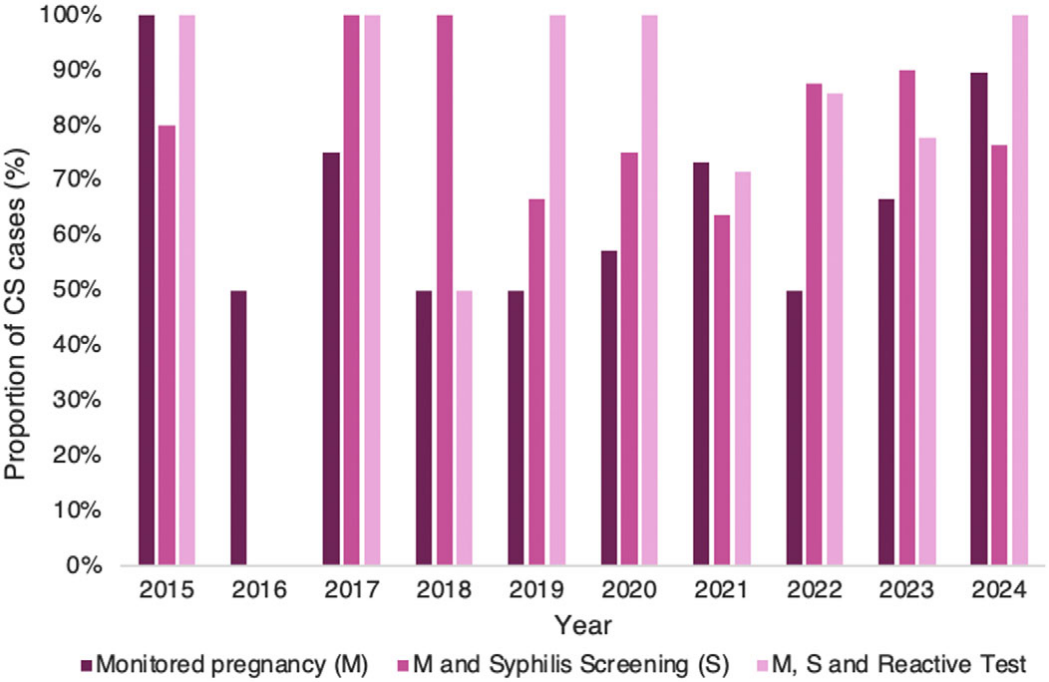
Proportion of confirmed congenital syphilis cases with monitored pregnancies (M); the subset of these cases that underwent antenatal syphilis screening in the 1st and 3rd trimesters (S), and the proportion of these with a reactive test result, by year, from 2015 to 2024. Figure 2. long description.

Between 2015 and 2024, 67.7% of the mothers of confirmed CS cases had their pregnancies monitored (Figure 1). However, 21.2% of pregnancies were not monitored. The lack of pregnancy monitoring was particularly relevant in 2016, 2018, 2019, and 2022, when only half of the mothers of confirmed CS cases had monitored pregnancies (Figure 2).

Of the pregnancies considered monitored, 77.6% included antenatal screening in the first and third trimesters, while in 11.9% of cases, it was either unknown or unreported whether screening occurred. In 2017 and 2018, all monitored pregnancies of confirmed CS cases underwent antenatal screening in both trimesters. Regarding the screening results, 88.5% of the screenings were reactive.

**Figure 3.**
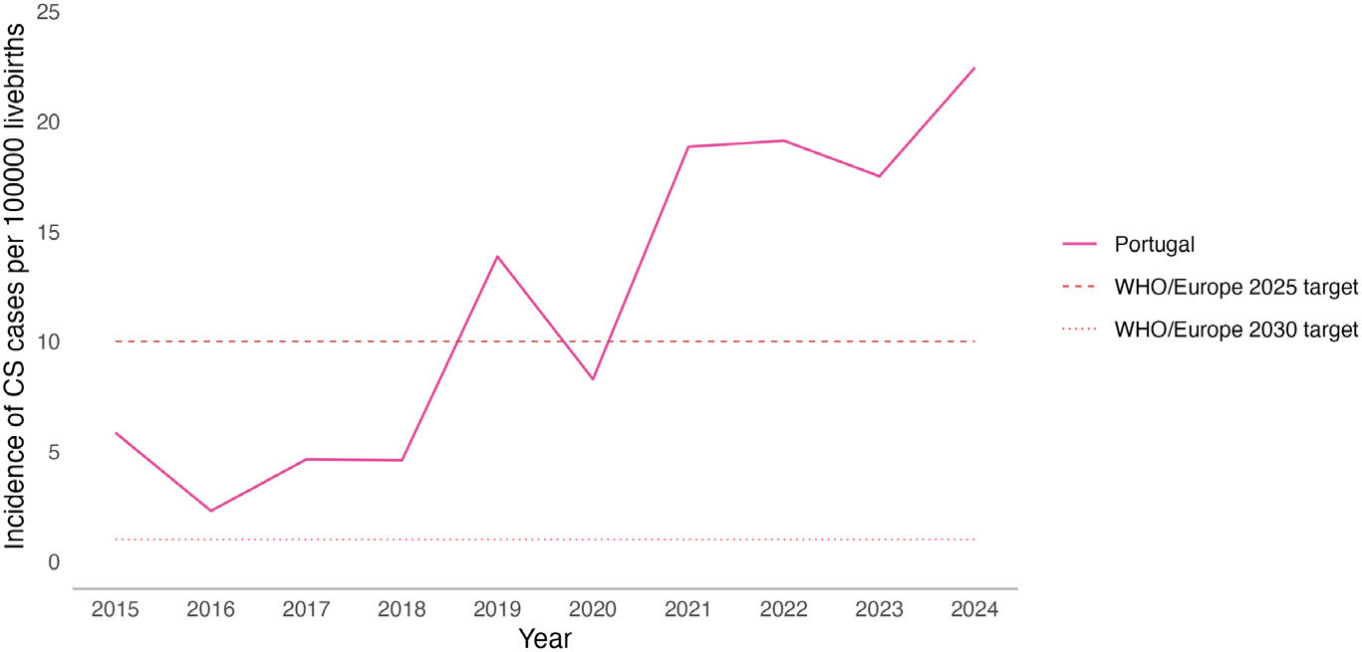
Incidence of confirmed congenital syphilis cases per 100000 live births in Portugal, from 2015 to 2024, with comparison with the WHO/Europe targets for the elimination of vertical transmission of syphilis set for 2025 and 2030. Figure 3. long description.

From 2016 to 2024, the incidence of confirmed CS cases per 100000 live births in Portugal increased approximately eightfold. The incidence doubled from 2015 to 2019, reaching 13.9 cases per 100000 live births that year. In 2020, at the onset of the SARS-CoV-2/COVID-19 pandemic, CS incidence decreased to 8.3 cases per 100000 live births. From 2021 to 2024, the trend reversed and reached a rate of 22.4 cases per 100000 live births in 2024, above the WHO Regional Office for Europe targets for 2025 and 2030, respectively (Figure 3).

**Figure 4.**
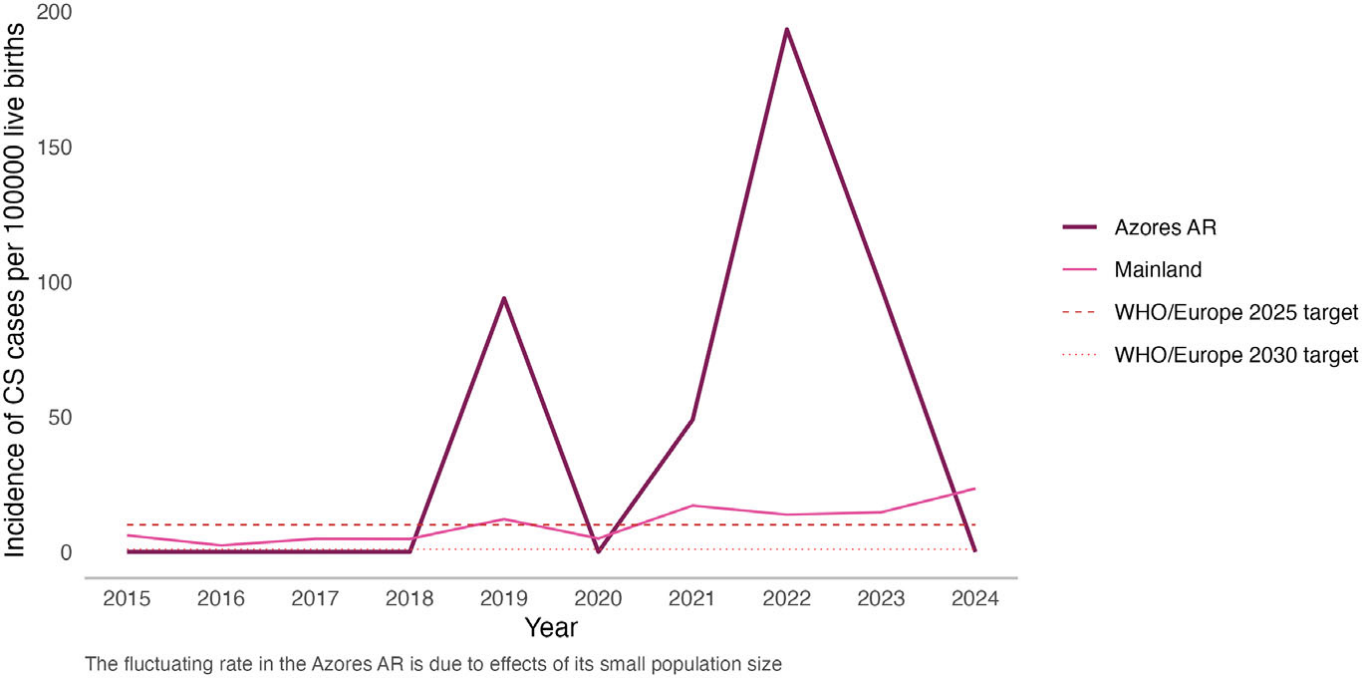
Incidence of confirmed congenital syphilis cases per 100000 live births in Portugal by NUTS I region, from 2015 to 2024, with comparison to the WHO/Europe targets for the elimination of vertical transmission of syphilis for 2025 and 2030. *Note*: No cases of CS were reported in Madeira AR during the study period. Figure 4. long description.

Marked differences were observed between mainland Portugal and the Azores and Madeira Autonomous Regions (AAR and MAR, respectively), from 2015 to 2024 (Figure 4). Although the absolute number of cases in AAR was lower, its incidence exceeded that of mainland Portugal in 2019 and again from 2021 to 2023. In those years, both AAR and mainland Portugal had an incidence higher than the WHO Regional Office for Europe target for 2025. In 2022, AAR recorded an incidence rate of 193.4 cases per 100000 live births – 9.7 times higher than that of the mainland. No cases of CS were reported in MAR during the study period.

## Discussion

Although historical Portuguese surveillance reports indicated a declining incidence of CS until 2016 [21], this trend reversed with an eightfold increase from 2016 to 2024. A temporary decrease occurred in 2020, likely related with the impact of the SARS-CoV-2/COVID-19 pandemic. However, by 2022, the incidence had resumed its pre-pandemic upward trajectory. The changes introduced in the EU case definition in 2018, particularly the removal of the requirement for IgM testing and the clarification of laboratory and epidemiological criteria, may have had an impact on surveillance from 2019 onwards, potentially influencing both the number of notifications.

This recent rise in CS incidence in Portugal does not appear to be explained by changes in surveillance sensitivity or clinical guidelines, which have remained stable throughout the study period [9, 10]. The higher incidence observed in the AAR may reflect a combination of region-specific factors, as previously discussed in a study conducted at one of the AAR’s public hospitals [22]. These factors include a limited number of obstetrics and gynaecology (OB-GYN) specialists, a high demand for OB-GYN hospital services, which are only available in certain islands, and a rise in syphilis cases, particularly among young mothers and marginalized groups within the AAR [22].

CS can be prevented with appropriate antenatal care and maternal treatment [7]. Therefore, the observed increase in Portugal is particularly concerning. The findings of this study indicated that a substantial proportion of CS cases were linked to unmonitored pregnancies. Among those classified as monitored pregnancies, 77.6% performed antenatal syphilis screening in both trimesters, which is recommended in Portuguese Standard for all pregnancies [9, 10]. A U.S.-based study reported comparable findings, with 36.8% of CS cases involving mothers who had either untimely or no documented syphilis testing during pregnancy [23]. Since two-thirds of confirmed CS cases in Portugal between 2015 and 2024 occurred in monitored pregnancies, there are potential fragilities in the clinical management of maternal syphilis; contributing factors may include absence or refusal of treatment, poor adherence to treatment regimens, particularly in cases of late latent syphilis or syphilis of unknown stage, delayed initiation of therapy, or reinfection following treatment. Reinfection can be linked to the lack of diagnosis and treatment of sexual partners, underscoring the essential role of partner management in preventing CS. Evidence from studies conducted in the United States, Italy, and Portugal have demonstrated that a substantial proportion of mothers of CS cases received inadequate, undocumented, or no treatment during pregnancy [22–25]. Furthermore, information pertaining to partner diagnosis and treatment is also poorly documented [22, 25]. The absence of treatment-related variables (including prescribed treatment, timing of treatment, and treatment completeness), together with the lack of information on partner(s)’ syphilis status and corresponding treatment in the Portuguese epidemiological surveillance system limits the ability to identify missed prevention opportunities and remains a barrier to achieving true elimination of CS. Additionally, although pregnant women diagnosed with syphilis can be identified when manually recorded, the system lacks automatic identification, limiting the evaluation of screening and treatment effectiveness. Future studies focused on linking epidemiological data with clinical data from hospitals and primary care to fill these knowledge gaps and strengthened protocols to ensure timely and complete treatment of pregnant people and their partners are needed.

Although direct identification of TP is highly specific, only a minority of cases were diagnosed through this method possibly due to its limited feasibility in routine clinical settings. In opposition, the availability and the use of specific IgM testing to diagnose CS is of particular importance, as general treponemal antibodies are not useful in diagnosing congenital syphilis, since maternal IgG antibodies cross the placental barrier and can persist in the newborn regardless of active infection [3, 26].

As a sentinel indicator of maternal healthcare quality, the upward trend of CS cases in Portugal underscores the need for further investigation into the underlying risk factors associated with CS and the provision of maternal healthcare in Portugal. The rising incidence of syphilis in Portugal and across the EU/EEA highlights the importance of analysing surveillance data to identify groups at higher risk of acquiring syphilis and to explore associations with CS, as has been done in other countries [27]. However, the current surveillance practices in Portugal, which rely on pseudonymized notifications for public health services, limit the ability to interrupt transmission chains, manage sexual partners, implement targeted prevention and control strategies, and effectively link CS cases to their corresponding maternal syphilis case. Mapping these risk-factors can help to improve the coverage and quality of maternal healthcare for all women residing in Portugal.

Our study has several limitations. Firstly, given the descriptive design, it is not possible to establish causal relationships or identify risk-factors for the occurrence of CS. The analysis is limited to describing the estimated incidence and maternal and infant demographic characteristics of CS cases reported through our surveillance system. Secondly, because the epidemiological survey does not capture the number of antenatal care visits in the epidemiological survey, the classification of a pregnancy as monitored is based on the clinical judgement of the reporting physician and/or the public health team responsible for the case investigation. Consequently, it is possible that the number of CS cases is underestimated. Importantly, the lower proportion of monitored pregnancies observed among CS cases does not imply reduced antenatal care coverage in the general population, nor does it indicate that Portugal is failing to meet the WHO target of 95% coverage for elimination efforts. Thirdly, the lack of specific questions regarding the mother and partner(s) treatment, and follow-up, reflects the fact that such information lies outside the scope of the epidemiological surveillance system. In addition, inconsistencies in how the antenatal screening question is interpreted – particularly the inability to report by trimester, further limit the system’s capacity to capture key elements relevant to CS prevention. An additional limitation pertains to incompleteness of maternal nationality data. From 2015 to 2019, the nationality of the mother was either unknown or not reported in the majority of cases, potentially influencing the observed results.

Other potential sources of information biases may have influenced the findings of this study. Underreporting may have led to an underestimation of the true national incidence of CS. Incomplete answers to optional questions of epidemiological surveys may lead to the mischaracterization of infants and their mothers, although updates to SINAVE during the study period have improved data completeness. Furthermore, cases diagnosed during 2024 but reported after 31 March 2025, were not included in the analysis, potentially leading to an undercount of cases.

Moreover, the EU case definition is less sensitive than the WHO case definition, a discrepancy previously documented in the literature [16, 18, 28]. This difference may contribute to an underestimation of the true burden of the disease. Additionally, stillbirths and foetal deaths due to CS are not reported through SINAVE, further compounding the underreporting of total cases. As a result, the actual burden of CS in Portugal and across the EU/EEA may be higher.

Portugal faces ongoing challenges in meeting the targets set by the WHO Regional Office for Europe for the elimination of vertical transmission of syphilis by 2025 and 2030. This underscores the importance of reinforcing efforts to enhance the prevention of vertical transmission within the national healthcare framework. Evidence from this and future studies will be essential for informing health policies and strengthening national programmes in STI, HIV, sexual and reproductive health, and child and youth health, while guiding improvements to existing interventions and the development of new strategies for key populations in Portugal. The findings of this study provide useful insight into where gaps in antenatal care most commonly occur and may support efforts to improve the early detection and management of maternal syphilis. Researchers can use these results to prioritize investigations into missed prevention opportunities and to refine strategies for identifying populations at higher risk of CS. For policy makers, the evidence points to areas where surveillance and programme implementation can be strengthened, helping to guide more targeted interventions towards the elimination of CS. To facilitate early detection and timely treatment of syphilis in pregnancy, and to prevent CS, it is strongly recommended to raise awareness among health professionals regarding the resurgence of syphilis and CS across the EU/EEA. Reviewing and updating guidelines for cases and partner management, alongside with evaluating the surveillance system and monitoring data quality, may further strengthen the national response. Ultimately, meaningful investment in these programmes, commitment to prioritizing reproductive health, maternal health, and child health and the reinforcement of health systems is important to mitigate this pressing public health issue.

## Data Availability

The cases were submitted to TESSy (The European Surveillance System) according to the established schedule. The database of live births in Portugal, categorized by year and NUTS I region, is maintained by the Statistics Portugal. This dataset provides detailed statistics on live births, including information categorized by the mother’s place of residence and other demographic factors. This information is accessible through the Statistics Portugal’s official website or the open data portal.

## Long descriptions

### Table 1. Long description

The table is organized into two columns: Variables and Number of confirmed C S cases (percentage).

* Sex: Male 52 (52.5%), Female 47 (47.5%).

* Age group: Less than 1 month 64 (64.6%), 1 to 12 months 34 (34.3%), Greater than 12 months 1 (1.0%).

* N U T S I region: Mainland 84 (90.3%), Azores A R 9 (9.7%), Madeira A R 0 (0.0%).

* Symptoms (Total): Yes 51 (51.5%), No 45 (45.5%), Unknown 3 (3.0%).

* Symptoms by age: For those less than 1 month, 46.9% had symptoms; for 1 to 12 months, 58.8% had symptoms; for greater than 12 months, 100% (1 case) had symptoms.

* Specific Clinical Symptoms (Yes responses): Jaundice 29.3%, Hepatosplenomegaly 24.2%, Anaemia 23.2%, Mucocutaneous lesions 18.2%, Central nervous involvement 15.2%, Pseudoparalysis 4.0%, Persistent rhinitis 3.0%, Malnutrition 3.0%, Condyloma lata 1.0%, Nephrotic syndrome 0%.

* Laboratory Demonstration of T. pallidum: Dark field microscopy was positive in 3.0% of cases; D F A - T P was positive in 1.0% of cases; Detection of T. pallidum-specific I g M and a reactive non-treponemal test in the child’s serum was positive in 98.0% of cases.

Navigate back to Table 1..

### Table 2. Long description

The table presents data for 99 confirmed C S cases, categorized by several maternal variables.

* Mothers age group: The largest group is 20 to 29 years with 40 cases (40.4 percent), followed by 30 to 39 years with 31 cases (31.3 percent), less than 20 years with 11 cases (11.1 percent), and greater than 40 years with 4 cases (4.0 percent). 13 cases (13.1 percent) are unknown.

* Mothers nationality: 48 mothers (48.5 percent) are from Portugal, 21 (21.2 percent) are foreign, and 30 (30.3 percent) are unknown.

* Syphilis stage: The majority are unknown at 55 cases (55.6 percent). Known stages include Early latent (16 cases, 16.2 percent), Primary (13 cases, 13.1 percent), Late latent (12 cases, 12.1 percent), and Secondary (3 cases, 3.0 percent).

* Previously given birth to a child diagnosed with C S: 70 mothers (72.2 percent) answered no, 2 (2.1 percent) answered yes, and 25 (25.8 percent) are unknown.

* Ongoing I D U (injecting drug use): 63 mothers (65.6 percent) answered no, 1 (1.0 percent) answered yes, and 32 (33.3 percent) are unknown.

* Previous history of I D U: 59 mothers (61.5 percent) answered no, 3 (3.1 percent) answered yes, and 34 (35.4 percent) are unknown.

Navigate back to Table 2..

### Figure 1. Long description

A stacked bar graph with a vertical Y axis labeled Proportion of C S cases percent, ranging from 0 to 100 percent in increments of 10. The horizontal X axis contains three categories. A legend at the bottom identifies three colors: dark purple for Yes, medium gray for No, and light gray for Unknown forward slash Not reported.

* The first bar, Monitored pregnancy M, shows approximately 68 percent Yes, 21 percent No, and 11 percent Unknown.

* The second bar, M and Syphilis Screening S, shows approximately 78 percent Yes, 10 percent No, and 12 percent Unknown.

* The third bar, M, S and Reactive Test, shows approximately 88 percent Yes, 10 percent No, and 2 percent Unknown.

Light purple shaded areas connect the top of the Yes sections between the bars, illustrating an upward trend in the proportion of positive responses as the criteria narrow from general monitoring to reactive testing.

Navigate back to Figure 1..

### Figure 2. Long description

The vertical y-axis represents the Proportion of C S cases in percent, ranging from 0 to 100 percent in increments of 10. The horizontal x-axis represents the Year from 2015 to 2024. Each year contains a cluster of three bars.

* The dark purple bar represents Monitored pregnancy M.

* The medium pink bar represents M and Syphilis Screening S.

* The light pink bar represents M, S and Reactive Test.

Data trends by year:

* 2015: M is at 100 percent, S is at 80 percent, and Reactive Test is at 100 percent.

* 2016: Only M is shown at 50 percent; other bars are absent.

* 2017: M is at 75 percent, while S and Reactive Test are both at 100 percent.

* 2018: M is at 50 percent, S is at 100 percent, and Reactive Test is at 50 percent.

* 2019: M is at 50 percent, S is at 67 percent, and Reactive Test is at 100 percent.

* 2020: M is at 57 percent, S is at 75 percent, and Reactive Test is at 100 percent.

* 2021: M is at 73 percent, S is at 64 percent, and Reactive Test is at 71 percent.

* 2022: M is at 50 percent, S is at 88 percent, and Reactive Test is at 86 percent.

* 2023: M is at 67 percent, S is at 90 percent, and Reactive Test is at 78 percent.

* 2024: M is at 90 percent, S is at 76 percent, and Reactive Test is at 100 percent.

Navigate back to Figure 2..

### Figure 3. Long description

The X-axis is labeled Year and ranges from 2015 to 2024. The Y-axis is labeled Incidence of C S cases per 100000 live births and ranges from 0 to 25.

* The data for Portugal is represented by a solid pink line. It begins at approximately 6 in 2015, drops to its lowest point of about 2.5 in 2016, then fluctuates upward to 14 in 2019. After a sharp dip to 8 in 2020, the incidence rises steeply to 19 in 2021 and 2022, followed by a slight dip in 2023 and a final peak at approximately 22.5 in 2024.

* A dashed horizontal pink line represents the W H O forward slash Europe 2025 target, set at a constant value of 10. The Portugal data line has remained consistently above this target since 2021.

* A dotted horizontal pink line represents the W H O forward slash Europe 2030 target, set at a constant value of approximately 1. The Portugal data line remains significantly above this target for the entire duration shown.

* A legend on the right side identifies the solid line as Portugal, the dashed line as W H O forward slash Europe 2025 target, and the dotted line as W H O forward slash Europe 2030 target.

Navigate back to Figure 3..

### Figure 4. Long description

The X-axis represents the Year from 2015 to 2024. The Y-axis represents the Incidence of C S cases per 100000 live births, ranging from 0 to 200.

* Azores A R (dark purple line): Remains at 0 from 2015 to 2018. It spikes to approximately 95 in 2019, drops back to 0 in 2020, rises to 50 in 2021, and reaches a peak of nearly 200 in 2022. It then declines to roughly 100 in 2023 and returns to 0 in 2024.

* Mainland (light pink line): Shows a relatively stable but slightly increasing trend. It starts near 7 in 2015, fluctuates between 3 and 13 until 2020, and then shows a gradual upward trend reaching approximately 24 by 2024.

* W H O forward slash Europe 2025 target: A horizontal dashed pink line fixed at an incidence of 10.

* W H O forward slash Europe 2030 target: A horizontal dotted pink line fixed at an incidence of 1.

A note at the bottom states that the fluctuating rate in the Azores A R is due to effects of its small population size.

Navigate back to Figure 4..
